# Preconception and Pregnancy Nutrition Support for Women with a History of Bariatric Surgery: A Mixed-Methods Survey of Healthcare Professionals in the UK

**DOI:** 10.3390/nu15204415

**Published:** 2023-10-18

**Authors:** Zainab Akhter, Judith Rankin, Alice Shackford-Alizart, Roger Ackroyd, Roland Devlieger, Nicola Heslehurst

**Affiliations:** 1 Population Health Sciences Institute, Newcastle University, Newcastle upon Tyne NE2 4AX, UKnicola.heslehurst@newcastle.ac.uk (N.H.); 2 Department of Surgery, Sheffield Teaching Hospitals, Sheffield S10 2JF, UK; 3 Department of Obstetrics and Gynaecology, University Hospitals Leuven, 3000 Leuven, Belgium

**Keywords:** pregnancy, preconception, bariatric surgery, obesity, nutrition, counselling

## Abstract

Preconception bariatric surgery improves obesity-related maternal pregnancy complications but may reduce the absorption of nutrients required for healthy fetal growth and development. Women who receive preconception nutritional support after bariatric surgery are less likely to have adverse pregnancy outcomes. This study aimed to investigate the provision of preconception and pregnancy-specific nutritional support for women having bariatric surgery in the UK. A mixed-methods survey was distributed to healthcare professionals working in obesity or maternity services between December 2018 and October 2019. We collected both quantitative and qualitative data which were analysed using a mixed-methods approach. We received 135 responses from online (*n* = 99) and postal (*n* = 36) questionnaires. Only 45% of participants reported being ‘very familiar’ with the preconception/pregnancy nutritional needs of this population. Barriers to providing nutritional support included: a lack of resources and time; poor communication both across services and with women; not having contact with women preconception; and a lack of information and guidance. Respondents felt that dietitians have the expertise in nutrition necessary to provide support; however, GPs and midwives have the most frequent patient access post-surgery, both before and during pregnancy. Optimal preconception and pregnancy-related nutritional support requires multidisciplinary care pre- and post-surgery, and healthcare professionals require training and guidance to inform practice.

## 1. Introduction

The majority of patients undergoing bariatric surgery for obesity are women of reproductive age [1]. Bariatric surgery, also known as weight loss surgery or metabolic surgery, is a procedure which involves alteration of the digestive system to aid in weight loss and the improvement or resolution of metabolic conditions [2]. Undergoing bariatric surgery prior to pregnancy can improve comorbidities associated with obesity, such as subfertility, gestational diabetes, and hypertension [3,4]. However, bariatric surgery patients are at risk of having poor nutritional status due to reduced nutritional intake and absorption [5]. The physiological changes during pregnancy, the increased nutritional demand, and pregnancy-related nausea and vomiting can exacerbate nutritional deficiencies and may have negative implications on fetal growth and development [6]. The risk of adverse perinatal outcomes, such as small-for-gestational-age (SGA) babies, are higher for women who have undergone bariatric procedures which bypass nutrient absorption sites in the intestine, such as gastric bypass (OR 2.72, 95% CI 2.32–3.20), compared to women without bariatric surgery [7]. A cohort study of women with a history of bariatric surgery found that 82% had a diet requiring improvement during pregnancy [8]. Women who have had bariatric surgery that report receiving preconception nutritional advice are significantly less likely to have a SGA baby than those who report not receiving advice (OR 0.15, 95% CI 0.04–0.55) [9]. These data demonstrate the need to ensure that preconception nutritional support is routinely provided to women of reproductive age who undergo bariatric surgery procedures, to optimise fetal development and improve pregnancy outcomes. However, the combination of rapid weight loss, endocrine changes, and malabsorption of oral contraceptives following bariatric surgery results in improved fertility immediately post-operatively; therefore, women are at risk of an unexpected pregnancy and limited opportunity for preconception planning [10,11].

In England, National Institute for Health and Care Excellence (NICE) guidelines state that bariatric surgery patients are required to have regular, specialist post-operative dietetic monitoring for a minimum for two years and are recommended to take lifelong nutritional supplements [12]. However, there is no guidance recommending discussions or referrals—either pre- or post-operatively—that relate to preconception, contraception, or pregnancy. Additionally, NICE guidelines for antenatal care do not identify women with a history of bariatric surgery as higher risk and requiring additional care, unlike other UK and European societies such as the British Obesity and Metabolic Surgery Society and the Royal College of Obstetrics and Gynaecology [6,13,14,15]. Education and counselling for prospective bariatric surgery patients are not currently standardised across the UK, with information provided often being based on the clinical experience of the team [16]. The absence of any national consensus on this topic warranted an investigation of health professionals’ practice. The aim of this study was to investigate the preconception and pregnancy-specific nutritional support provided for women preparing for, or with a history of, bariatric surgery. We also explored the barriers to support provision, and any requirements healthcare professionals felt were needed to support their practice.

## 2. Materials and Methods

A mixed-methods survey was designed, targeting healthcare professionals working in obesity or maternity services in England and Wales (Figure S1). Self-administered questionnaires are useful tools for collecting data on human phenomena such as attributes, knowledge, behaviour, attitudes, experiences, and beliefs [17]. We designed the questionnaire using a mixture of quantitative and qualitative questions to allow detailed answers while also allowing completion in under ten minutes to maximise questionnaire completion rates among busy healthcare professionals [18]. Quantitative questions explored healthcare professionals’ understanding, level of awareness, and their opinions on ideal practice. Qualitative questions enabled participants to provide further detail on different aspects and specifics of their practice, and the barriers to practice they faced. The questionnaire was initially piloted among four participants to establish validity. Online links to the questionnaire were distributed using relevant society mailing lists such as the Association for the Study of Obesity (ASO), the British Dietetics Association (BDA), and the British Obesity and Metabolic Surgery Society (BOMSS), as well as on social media networks Facebook, Twitter, and Instagram between December 2018 and March 2019. The questionnaire was distributed by a known contact, if possible, to avoid being misconstrued as junk mail. Snowball sampling was encouraged to facilitate wider distribution. To supplement the online strategy, paper-based questionnaire packs were posted out to 120 NHS Trusts and 20 private hospitals which provided maternity or bariatric services in England and Wales in October 2019. The packs contained multiple paper copies of the questionnaire; pre-paid return envelopes; participant information sheets; a short link and QR code to the online survey; and a recruitment poster to be placed in communal space. The data from questionnaires returned by post were entered into the online survey by a member of the research team (Z.A). Responses from participants were excluded if they were not a healthcare professional working in obesity or maternity care.

All data collected were kept confidential and used only for the purposes of this research study. Informed consent from participants was obtained before the beginning of the survey, with participant information and a privacy notice displayed on screen and the option to disagree and exit. Quantitative analysis included absolute and relative frequencies for each response. Total percentages add up to over 100% for questions in which multiple answers could be selected. The quantitative data were managed and analysed in Stata IC 16.0. The qualitative data were analysed using the framework method, which is a systematic method involving familiarisation of data, development of a thematic framework, coding, charting, then final mapping and interpretation of the data [19]. Both inductive and deductive approaches to coding were used to ensure that the analysis explicitly addressed the study aim whilst also allowing the exploration of healthcare professionals’ personal views and experiences and enabling these to be incorporated into the framework. Examples of verbatim terms and quotes from respondents are presented in italics inside quotation marks for context. The qualitative data were stored and managed in NVivo 12. Both quantitative and qualitative data supplement each other and provide a wider context, therefore the results are reported in an integrated manner according to the study questions.

## 3. Results

A total of 137 responses were received and 135 were from people in healthcare professional roles eligible for inclusion in this study. Due to the survey distribution methods used, in particular social media and snowball sampling, the final reach and therefore response rate of the survey was unknown. Dietitians accounted for approximately one third of the respondents (31.9%) and midwives for one quarter (25.9%). The remainder of the responses were received from nurses (9.6%), GPs (7.4%), bariatric surgeons (6.7%), and obstetricians (6.0%). Respondents that selected ‘Other’ (12.6%) defined themselves as endocrinologists, nutritionists, physicians, or other tier 3 weight management service staff (Table 1). Respondents were from all regions of England and Wales, with a quarter of responses being from the North East (25.2%).

The results are presented in four thematic discussion sections: (1) healthcare professionals’ self-reported understanding of the preconception and pregnancy nutritional needs of women who have undergone bariatric surgery; (2) current provision of advice; (3) the barriers to providing advice; and (4) perspectives on who is best placed to provide advice, and when.

### 3.1. Understanding of Nutritional Needs

**Figure 1:**
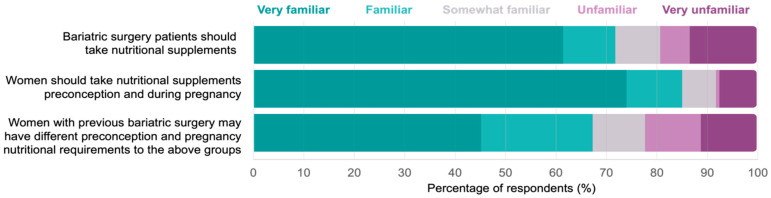
Healthcare professionals’ familiarity with three statements regarding nutritional supplementation in bariatric surgery patients, women during preconception and pregnancy, and women with a history of bariatric surgery during preconception and pregnancy.

Healthcare professionals ranked their familiarity with the three statements regarding nutritional supplement requirements for women: (1) after bariatric surgery, (2) during preconception and pregnancy, and (3) with a history of bariatric surgery during preconception and pregnancy (Figure 1). Respondents were most familiar with supplement requirements for statements 1 and 2, with less than half (n = 61, 45.2%) being ‘very familiar’ with the requirements for statement 3.

In the qualitative data, respondents reported key nutritional information for female bariatric surgery patients thinking of conceiving or already pregnant, ranging from general pregnancy recommendations—such as eating a balanced diet and taking a multivitamin—to more specialised advice, such as taking a 5 mg folic acid supplement.

‘*Folic acid-probable increased requirement if [patients] still have high BMI and due to malabsorption (especially for bypass)*.’

—Participant 7, Dietitian

### 3.2. Provision of Advice

Over a quarter (27.4%) of healthcare professionals reported offering no nutritional advice to women who are planning, or have had, bariatric surgery, specifically regarding preconception and pregnancy (Table 2). Half of the respondents reported providing ‘Detailed’ or ‘Some’ level of advice.

Out of the respondents that provided advice, most reported offering nutritional advice either during pregnancy (57.1%) or at the point of surgery (52%). Fewer offered nutritional advice before surgery (41.8%) or preconception (36.7%). Information sources for healthcare professionals were primarily from specialist society guidelines such as BOMSS and RCOG (67.3%), followed by local guidelines (36.7%) and research publications (36.7%).

In the open responses, healthcare professionals reported delivering advice verbally, most commonly one to one in a clinic, but also either on the phone or in group sessions as part of pre-operative education. Some provided written advice or leaflets in addition to verbal advice if these were available at their hospital or signposted to reputable websites. The concept of providing information in a written, individualised format was mentioned, such as letters or care plans sent to women’s homes, to midwives, and to GPs, so that they were aware of their specialised nutritional requirements. The content of the advice provided was mostly dietary advice and micronutrient supplementation. Healthcare professionals recommended referral to specialist dietitians and regular nutritional monitoring throughout pregnancy.

‘*Verbal advice including advice on diet, supplementation, nutritional deficiencies (bloods will be routinely monitored). Letter detailing advice to GP, pt [patient], and midwife (for pregnant bariatric patients) will be sent as and when required*.’

—Participant 119, Dietitian

There was a lack of consistency between healthcare professionals relating to their advice on surgery-to-conception intervals, which ranged from 6 to 18+ months (Table 3). ‘Other’ durations of conception intervals described included: at least two years; advice dependent on the type of surgery (less time for gastric band, more for gastric bypass and sleeve gastrectomy); or waiting for the stabilisation of weight regardless of the length of time since surgery.

### 3.3. Barriers to Providing Advice

A lack of information was commonly reported by respondents as a barrier to providing nutritional support to women regarding pregnancy after bariatric surgery. Healthcare professionals wanted to support women with their nutrition but were unsure of what advice should be provided and where to access information to inform practice, stating that they require formal education, training, and guidelines. GPs also described how they were dependent on information provided in hospital letters from the bariatric service to help them support this group of women.

Challenges with communication around pregnancy intentions was raised as a barrier to the provision of preconception nutritional care. Prior to surgery and after surgery, healthcare professionals reported feeling ‘*awkward*’ or ‘*not confident enough*’ to bring up the subject of pregnancy. They noted that many women may have experienced sub-fertility related to their obesity status prior to surgery and may not wish to discuss pregnancy, or they assumed that the information is not relevant to them.

‘*Some women who have struggled with fertility have not ensured they are taking appropriate contraception at the time of surgery despite being advised to do so. I guess they feel it is highly unlikely to happen to them based on their previous experience*.’

—Participant 61, Nurse

‘*No information has been given to staff from specialist teams and sometimes clients are reluctant to tell you they have had the surgery—they don’t see it as something to be shared*.’

—Participant 70, Public Health Practitioner

Barriers to the provision of nutritional support relating to inconsistent contact, such as engagement with services, were reported. For example, respondents reported that there was sometimes low/non-attendance for post-surgery appointments, which was a barrier to providing nutritional support at this important stage for preconception support. Additionally, patients were discharged from bariatric pathways after the two-year follow up period, limiting the ability to provide longer-term preconception nutritional support. Healthcare professionals also reported feeling a ‘*disconnect between obesity services and maternity services*’ and felt that there should be more joined up, multidisciplinary care for women of reproductive age to avoid losing this group of women to follow up.

‘*We do rely on the patient letting us know they are pregnant or planning to get pregnant. They may be missed if they do not attend follow up or have been discharged from the service as over 2 years after their surgery*.’

—Participant 61, Nurse

‘*Access to women—services not joined up so may have left bariatric care when they get pregnant, may not think to tell midwives*.’

—Participant 14, Dietitian

The final barrier related to the amount of time and physical resources, or the lack thereof, available to the healthcare professionals. Respondents felt that, in the bariatric service, appointments were time-pressured and pregnancy-related nutritional support could be missed. Additionally, pre- and post-operative education were not standardised—which could result in inconsistency in the provision of support between patients—and leaflets were suggested as being useful if there was limited time for discussion. In the maternity service, healthcare professionals described how there are preconception clinics for some high-risk groups, such as women with diabetes, but not for bariatric surgery patients. Therefore, support was usually provided during pregnancy with less opportunity for preconception support. Finally, some healthcare professionals stated that their service did not allow prescriptions for supplements, and this was a barrier to practice as they could only recommend supplements for purchase and hope the women followed the advice.

‘*Time pressures in appointments mean [preconception/pregnancy advice provision] is low on priority list to mention unless patient asks specifically*.’

—Participant 43, Dietitian

### 3.4. Ideal Delivery of Nutritional Advice

Health professionals reported that they felt it was most appropriate to deliver support either before bariatric surgery is carried out (n = 63, 46.7%) or when women are seeking preconception support (n = 64, 47.4%). However, they also stated that all time points in the pathway are appropriate. The importance of providing preconception/pregnancy-related nutritional support before surgery was described as ensuring that the information was provided to all women before they could potentially be lost to follow-up and ensuring that women are fully aware of the impact of surgery on future pregnancies, the increased fertility, and preconception nutritional needs. Respondents stated that women’s knowledge of the benefits and risks of bariatric surgery on pregnancy could influence the decision of when they undergo the procedure, which procedure they undergo, or if they commit to it at all.

‘*It would be important for patients to understand whether surgery is a good option for them prior to referral as we recommend avoiding conception for 2 years post-op. They need to know benefits and risks of bariatric surgery prior to conception*.’

—Participant 36, Endocrinologist

However, there was also a viewpoint that women may not feel the information is relevant to them pre-operatively or might forget it due to the volume of information at that time relating to the surgery itself. Whereas if women received support preconception or during pregnancy, then ‘*the information will be fresh in their minds*’ and directly relevant to their current situation. The challenges with identifying women in the preconception stage stem from the fact that if they do not seek care, or if their pregnancy plans were not raised, then by the time they are pregnant/realise they are pregnant many key stages of embryonic development will have already occurred. Hence, there was a view that the only way to ensure women are well informed and prepared for a pregnancy was to deliver support at multiple points in the care pathway as *‘every opportunity is important*’ for discussion.

‘*They then have the information they need prior to conceiving. Pregnancy may be too late, especially in terms of fetal development and growth, and overall health of the woman*.’

—Participant 44, Midwife

Nearly all (n = 126, 93.3%) respondents felt that dietitians were best placed to provide preconception and pregnancy-related nutritional support due to their specialist knowledge on diet and supplementation. Respondents also suggested that other members of the immediate bariatric team such as endocrinologists, nurses, and surgeons had the correct training and knowledge to be able to provide the nutritional support and stressed the importance of a multidisciplinary approach. The importance of the multidisciplinary approach was also discussed in the context of health professionals with the most frequent opportunities to provide support. Healthcare professionals in maternity care, particularly midwives, were identified as having more frequent opportunities to discuss nutritional needs, and they often develop close relationships with women under their care, which allows more open conversations. However, a reliance on maternity teams would be too late for preconception nutritional support, and the professionals in routine obesity care and post-operative follow up teams could also be best placed to provide support. Finally, GPs and practice nurses were described as having the main preconception contact following discharge from bariatric teams; additionally, they maintain contact with the women for the rest of their lives, making primary care services the first point of healthcare contact. Primary care teams were also described as being the first healthcare professionals to know about a pregnancy, subsequently making the referral to maternity services—at this point they could also re-refer the woman to the bariatric team for more specialised support if required.

‘*Midwives have the access to women but lack the time or training to deliver. Dietitians can have the specialist knowledge but do not necessarily see these women*!’

—Participant 14, Dietitian

‘*All [healthcare professionals] should be aware of the issues but the bariatric team will have most contact with these patients prior to pregnancy. I think the advice should be reinforced by multiple team members not just one person*.’

—Participant 50, Bariatric surgeon

## 4. Discussion

This study aimed to investigate healthcare professionals’ awareness and provision of preconception and pregnancy-specific nutritional support for women with a history of bariatric surgery, and the barriers to providing support. The majority of healthcare professionals felt at least somewhat familiar with how preconception and pregnancy nutritional needs are different in women with previous bariatric surgery compared to the general population. However, only half of the healthcare professionals surveyed regularly provided this kind of support to bariatric surgery patients, and the preconception stage is often missed, with advice being provided during pregnancy. Barriers to providing nutritional support included a lack of time, resources, and information available to support practice. Respondents also reported barriers to contact and communication, such as poor attendance at follow-up appointments and pregnancy intentions not being disclosed. The overall perspective was that optimal preconception and pregnancy-related nutritional support requires a multidisciplinary approach both pre- and post-surgery.

The provision of advice regarding pregnancy at the pre-operative stage could be improved by overcoming the barriers reported in the survey. There are opportunities for healthcare professionals to invite discussions around pregnancy and fertility prior to surgery and post-operatively within the two-year follow-up period. These discussions are important, even if women feel like the information is not relevant to them, due to increased fertility post-surgery and issues relating to loss to follow up. This would also go some way to addressing the current lack of preconception nutrition information and the support void between bariatric and maternity services. A previous survey of contraception provision after bariatric surgery in England also found that discussions with patients around sexual and reproductive health were scarce both before and after bariatric surgery [20]. This study also reports on challenges with any singular time point or healthcare professional group providing preconception and pregnancy nutrition support, and the need for multidisciplinary, joined up care that capitalises on both expertise (e.g., dietitians and obesity specialists) and opportunity (e.g., primary care and maternity services).

Among the health professionals responding to this survey that reported providing some preconception and pregnancy-related nutritional support, most offered advice verbally. In the absence of in-person appointments, telephonic nutritional support provision has been shown to be successful in improving pregnancy outcomes after bariatric surgery [21]. The respondents of this study also described issues with information overload and retention. The need for written information and signposting to universally accessible online content in addition to verbal advice could help to address this. Some respondents reported that their NHS Trusts had leaflets with patient guidance available; however, these local-level resources could add to inconsistencies in the type of information provided, and whether someone receives this information depends on their service provider. It would be beneficial for UK healthcare services to have standardised national evidence-based guidelines to facilitate the same pre- and post-operative nutritional support for preconception and pregnancy across the country. In the US, the American College of Obstetrics and Gynecology has published guidelines for the care of pregnant women following bariatric surgery [22]. A survey of 106 US obstetricians found that only 36% always follow these guidelines, which indicates that not only is the availability of guidelines important, but also accessibility and implementation support to overcome any barriers to practice [23]. In our study, the need for guidelines was described by respondents, alongside the need for formal education and training in this area of practice. Guideline implementation challenges are likely to also relate to the lack of joined up care between bariatric and maternity services, as identified by the health professionals participating in this study. Improvements in communication and shared practice between bariatric surgery and women’s healthcare is required, to facilitate the provision of preconception and pregnancy-related nutritional support across the entire care pathway, so that the required care to improve maternal and infant health can be provided.

There are strengths and limitations to this research. The sample size of some health professional groups is small, and we are not able to fully assess the response rate due to methodological strategies of using social media for recruitment. However, a survey of this nature has never been completed before in the UK, and the variety of healthcare professionals spanning a range of practice settings and locations provides multiple perspectives to inform future research, practice, and policy needs. One of the limitations with questionnaires is that they are susceptible to bias, especially those with low response rates. The method of using questionnaires rather than interviews is more practical for larger populations and widespread distribution in terms of both cost and feasibility; however, it meant that the responses could not be followed up. Despite these challenges, the survey responses provided a novel understanding of the existing provision of advice in routine practice and the barriers to practice, as well as helping to inform areas for improving service provision.

## 5. Conclusions

This study provides a valuable insight into the provision of preconception and pregnancy-related nutritional support for women planning to have bariatric surgery in England and Wales. Previous studies have shown that preconception nutritional advice and support can improve infant health outcomes after bariatric surgery. Although some healthcare professionals reported familiarity with the nutritional needs of this population, there were many barriers to implementing support. In the absence of national consensus or guidelines, the type, amount, and content of support women receive is linked to the knowledge of their service providers, which will result in inequalities in the provision of evidence-based care. Having improved evidence-based guidance and resources is a strategy that will enable healthcare professionals to provide specialised preconception and pregnancy nutrition support, which will ultimately improve pregnancy outcomes after bariatric surgery.

## Figures and Tables

**Table 1:** Participant demographics.

	N	%
**Healthcare professional role**		
Dietitian	43	31.9
Midwife	35	25.9
Nurse	13	9.6
General practitioner	10	7.4
Bariatric surgeon	9	6.7
Obstetrician	8	5.9
Other	17	12.6
**Location**		
North East England	34	25.2
London	21	15.6
South East England	18	13.3
Yorkshire & The Humber	15	11.1
West Midlands	13	9.6
South West England	10	7.4
North West England	9	6.7
East of England	8	5.9
East Midlands	4	3.0
Wales	3	2.2

**Table 2:** Provision of nutritional advice regarding preconception and pregnancy to women who are planning, or have had, bariatric surgery.

	N	%
Detailed	33	24.4
Some (it is discussed)	35	25.9
Varies from patient to patient	18	13.3
Little (briefly mentioned)	12	8.9
None	37	27.4

**Table 3:** Provision of advice regarding surgery-to-conception interval.

	N	%
**Recommendation to delay pregnancy**		
6 months	1	1.0
6–12 months	2	2.1
12–18 months	23	23.7
18+ months	45	46.4
No recommendation	5	5.2
Other	21	21.6

## Data Availability

The data presented in this study are available on request from the corresponding author. The data are not publicly available due to participant confidentiality.

## Supplementary Materials

The following supporting information can be downloaded at: https://www.mdpi.com/article/10.3390/nu15204415/s1, Figure S1: Questionnaire.
