# Supplementary material for: Preconception and Pregnancy Nutrition Support for Women with a History of Bariatric Surgery: A Mixed-Methods Survey of Healthcare Professionals in the UK
Source: Nutrients. 2023 Oct 18;15(20):4415. doi: 10.3390/nu15204415 (PMC10609851; doi:10.3390/nu15204415)
Supplement: Supplementary file 1 [file nutrients-15-04415-s001.zip › nutrients-2614611-supplementary.pdf]

Figure S1. Questionnaire

Newcastle University

Institute of Health & Society

A survey of the current preconception and pregnancy nutritional support provided to female bariatric surgery patients

Thank you for taking the time to consider taking part in this survey, it should take you no longer than 10 minutes to complete all questions.

The aim of this survey is to explore the nutritional support provided to female bariatric surgery patients regarding preconception/pregnancy and any barriers to providing support.

We invite you to complete this survey if you are a **health professional in England and Wales working in obesity, bariatric surgery, or maternity services.** For example: bariatric surgeons, endocrinologists, tier 3 and 4 weight management services, general practitioners, nurses, midwives, dietitians, obstetricians, etc.

This study is being carried out at the Institute of Health and Society, Newcastle University.

Research team: Zainab Akhter, Alice Shackford, and Dr Nicola Heslehurst

Contact details: z.akhter@newcastle.ac.uk

The study has been approved by the Newcastle University Faculty of Medical Science ethics committee. If you agree to take part in this survey, the data you provide will be anonymous and stored by Online Surveys under the university license. The raw data will only be handled by the research team for the purposes of this study.

The results from this survey will form student research projects and may be published in a peer reviewed journal and presented at a conference.

If you prefer, complete the survey online by typing the following in to your internet browser:

bit.do/nutrition-survey

Do you agree to the privacy notice above and consent to take part in the survey?

Please tick one answer only.

○ Agree

○ Disagree

Part 1 – Demographics

1. Please select your job role:

- ○ Bariatric surgeon
- ○ Dietitian
- ○ General practitioner
- ○ Nurse
- ○ Midwife
- ○ Gynaecologist
- ○ Obstetrician
- ○ Other: ____________________________

2. Which area of England & Wales do you work in?

- ○ North East England
- ○ North West England
- ○ Yorkshire & The Humber
- ○ East Midlands
- ○ West Midlands
- ○ East of England
- ○ London
- ○ South East England
- ○ South West England
- ○ Wales

3. When, in the pathways of care, do you interact with bariatric surgery patients?

Please select all that apply.

- ☐ Tier 3 weight management services (Pre-operative)
- ☐ Tier 4 weight management services (Bariatric surgery)
- ☐ Tier 3 weight management services (Post-operative)
- ☐ Preconception
- ☐ Pregnancy
- ☐ Other: ____________________________

4. Please name the service you work for:

Note: this question is only being asked to determine survey response rate - no data will be linked to any specific service in the analysis.

Part 2 – Understanding of nutritional needs

5. **Please tick the familiarity you have with each of these three statements:**

	Very unfamiliar	Unfamiliar	Somewhat familiar	Familiar	Very familiar
**(1)** Bariatric surgery patients should take nutritional supplements					
**(2)** Women should take nutritional supplements preconception and during pregnancy					
**(3)** Women with previous bariatric surgery may have different preconception and pregnancy nutritional requirements to the above groups					

6. **What do you think the key nutritional information would be for female bariatric surgery patients thinking of conceiving, or already pregnant?**

Part 3 - Nutritional support

7. **Do you currently offer any advice, specifically regarding preconception and pregnancy, to women who are planning or have had bariatric surgery?**

- ○ Detailed
- ○ Some (it is discussed)
- ○ Varies from patient to patient
- ○ Little (briefly mentioned)
- ○ None *please skip* ***Part 4***

Part 4 – Delivered nutritional support

Please skip this page (Part 4) if you answered 'None' to question 7

8. Where have you sourced this information?

Please select all that apply.

.☐ NICE guidelines
☐ Society guidelines e.g. RCOG/BOMSS
☐ Hospital specific guidelines
☐ Research publications
☐ Other: ___________________________

9. When, in the pathways of care, do you offer this advice?

Please select all that apply.

☐ Tier 3 weight management services (Pre-operative)
☐ Tier 4 weight management services (Bariatric surgery)
☐ Tier 3 weight management services (Post-operative)
☐ Preconception
☐ Pregnancy
☐ Other: ___________________________

10. What type of advice do you provide?

E.g. Format (verbal advice, leaflets) Content (dietary advice, supplementations) etc.

11. Do you advise women of reproductive age to wait before trying to conceive after bariatric surgery, and if yes, how long?

○ 6 months
○ 6-12 months
○ 12-18 months
○ 18+ months
○ No
○ Other: ___________________________

12. Do you provide any nutritional supplements specific to female bariatric surgery patients who are planning to get, or are, pregnant?

Part 5 – Ideal delivery of nutritional support

13. For the next three questions please consider if there any barriers in place to you providing nutritional support for women of reproductive age who are:

**(1)** Planning bariatric surgery	
**(2)** Have had bariatric surgery	
**(3)** Post-surgery preconception or pregnancy	

14. When, in the pathway of care, do you think it is most appropriate to provide nutritional support specific to preconception and pregnancy for female bariatric surgery patients?

Please select all that apply.

- ☐ Tier 3 weight management services (Pre-operative)
- ☐ Tier 4 weight management services (Bariatric surgery)
- ☐ Tier 3 weight management services (Post-operative)
- ☐ Preconception
- ☐ Pregnancy
- ☐ Other: ___________________________

15. Why do you think this is the most appropriate point?

16. Which health professionals do you think are best suited to provide this support?

Please select all that apply.

- ☐ Bariatric surgeon
- ☐ Dietitian
- ☐ General practitioner
- ☐ Nurse
- ☐ Midwife
- ☐ Gynaecologist
- ☐ Obstetrician
- ☐ Other: ___________________________

17. Why do you think these health professionals are best suited to provide this support?

Part 6 – Comments

Is there anything else you think is important about this research topic you would like to add?

Thank you for taking the time to complete this survey.

Please enclose your survey in the prepaid envelope provided addressed to:

Zainab Akhter
Institute of Health & Society
Newcastle University
Baddiley-Clark Building
Richardson Road
Newcastle upon Tyne
NE2 4AX
United Kingdom

If you have any further questions or comments please contact z.akhter@newcastle.ac.uk.
